# The High Affinity IgE Receptor FcεRI Is Expressed by Human Intestinal Epithelial Cells

**DOI:** 10.1371/journal.pone.0009023

**Published:** 2010-02-02

**Authors:** Eva Untersmayr, Giovanna Bises, Philipp Starkl, Charles L. Bevins, Otto Scheiner, George Boltz-Nitulescu, Fritz Wrba, Erika Jensen-Jarolim

**Affiliations:** 1 Department of Pathophysiology, Center of Pathophysiology, Infectiology and Immunology, Medical University Vienna, Vienna, Austria; 2 Department of Microbiology and Immunology, University of California Davis School of Medicine, Davis, California, United States of America; 3 Department of Pathology, Medical University Vienna, Vienna, Austria; Charité-Universitätsmedizin Berlin, Germany

## Abstract

**Background:**

IgE antibodies play a paramount role in the pathogenesis of various intestinal disorders. To gain insights in IgE-mediated pathophysiology of the gut, we investigated the expression of the high affinity IgE receptor FcεRI in human intestinal epithelium.

**Methodology/Principal Findings:**

FcεRI α-chain, as detected by immunohistochemistry, was positive in epithelial cells for eight of eleven (8/11) specimens from colon cancer patients and 5/11 patients with inflammation of the enteric mucosa. The FcεRIα positive epithelial cells co-expressed FcεRIγ, whereas with one exception, none of the samples was positive for the β-chain in the epithelial layer. The functionality of FcεRI was confirmed *in situ* by human IgE binding. In experiments with human intestinal tumor cell lines, subconfluent Caco-2/TC7 and HCT-8 cells were found to express the α- and γ-chains of FcεRI and to bind IgE, whereas confluent cells were negative for γ-chains.

**Conclusions/Significance:**

Our data provide the first evidence that the components of a functional FcεRI are *in vitro* expressed by the human intestinal epithelial cells depending on differentiation and, more importantly, *in situ* in epithelia of patients with colon cancer or gastrointestinal inflammations. Thus, a contribution of FcεRI either to immunosurveillance or pathophysiology of the intestinal epithelium is suggested.

## Introduction

Although immunoglobulins are important constituents of host defense in mucosal compartments [1], they have been ascribed opposing functions in various intestinal diseases. Increased levels of immunoglobulin E (IgE) have been found during parasite infection with a putative beneficial host defense function [2], [3]. In contrast, IgE plays a documented detrimental role in allergy. Significantly increased levels of IgE and anti-IgE autoantibodies might contribute also to the pathophysiology in Crohn's disease (CD) [4]. Interestingly, it has been suggested that food allergic reactions might be triggered as a consequence of gastrointestinal inflammation [5], [6]. Additionally, growing evidence points towards a participation of IgE in antibody-dependent tumoricidal activities [7]–[9].

IgE function depends on its interaction with effector cells via specific surface-receptors. The high affinity IgE receptor (FcεRI) is a multimeric cell-surface receptor, which binds the Fc domain of IgE with an affinity of 10^10^ M^−1^ [10]. The conformational change of the IgE constant region that occurs upon binding to FcεRI was proposed to contribute to the remarkably slow dissociation rate of receptor-bound IgE [11]. FcεRI has been so far detected on human mast cells, basophils, neutrophils, monocytes, macrophages, dendritic cells, Langerhans cells, eosinophils and platelets [12]. While the extracellular domain of the receptor α-chain carries the IgE binding site [13], the β- and γ-chains are involved in signal transduction [14], [15]. The αβγ_2_ tetramer is expressed in effector cells such as mast cells and basophils, and ligand-engagement leads to cell activation by a defined signaling cascade. In contrast, the αγ_2_ trimer participates in antigen presentation [16].

The low affinity IgE receptor (FcεRII/CD23) is a single chain glycoprotein with a molecular weight of 49 kDa [17]. In contrast to FcεRI, CD23 binds IgE with a significantly lower affinity (10^7^ M^−1^). CD23 was initially identified on B-lymphocytes [18] but subsequently also detected on various other cell types such as monocytes, macrophages, eosinophils and Langerhans cells [17], [19], [20]. Interestingly, CD23 is also expressed on intestinal epithelial cells where it is elevated in inflammatory conditions such as CD and food allergies [21]. An IgE/CD23-dependent, transepithelial shuttle mechanism, regulated by interleukin (IL)-4, has been described, which mediates transport of intact food antigens [22]–[24].

Besides FcεRI and FcεRII/CD23, the IgE-binding protein (εBP, Galectin-3) also specifically interacts with IgE [25]. Due to its wide tissue distribution and expression on various cell types [26], a multifunctional role in cell growth regulation, cell adhesion and tumor metastases, among others, was suggested [27]–[29]. The intestinal distribution pattern of εBP is well established and it has been shown that it is downregulated in inflammation, whereas an elevated expression in colon cancer influences the neoplastic progression [30], [31].

The presence of CD23 and εBP on intestinal epithelia is well documented, and functional studies have supported their biological importance. However, since no data were available concerning expression of FcεRI on enterocytes to date, we screened the intestinal mucosa of patients with gastrointestinal pathologies and controls, as well as intestinal epithelial cell lines for FcεRI expression. Herein, we report that both FcεRI α- and γ-chains are expressed by intestinal epithelial cells, while FcεRI β-chain could only be detected in the subepithelial stroma. The IgE binding found in α- and γ-chain positive tissues indicates the presence of a functional trimeric receptor FcεRIαγ_2_ in human intestine, which could contribute to IgE-mediated pathophysiology of the gut.

## Results

### The High Affinity IgE Receptor FcεRI α- and γ-Chains Are Expressed on the Epithelium of Human Intestinal Tissue

**Figure 1:**
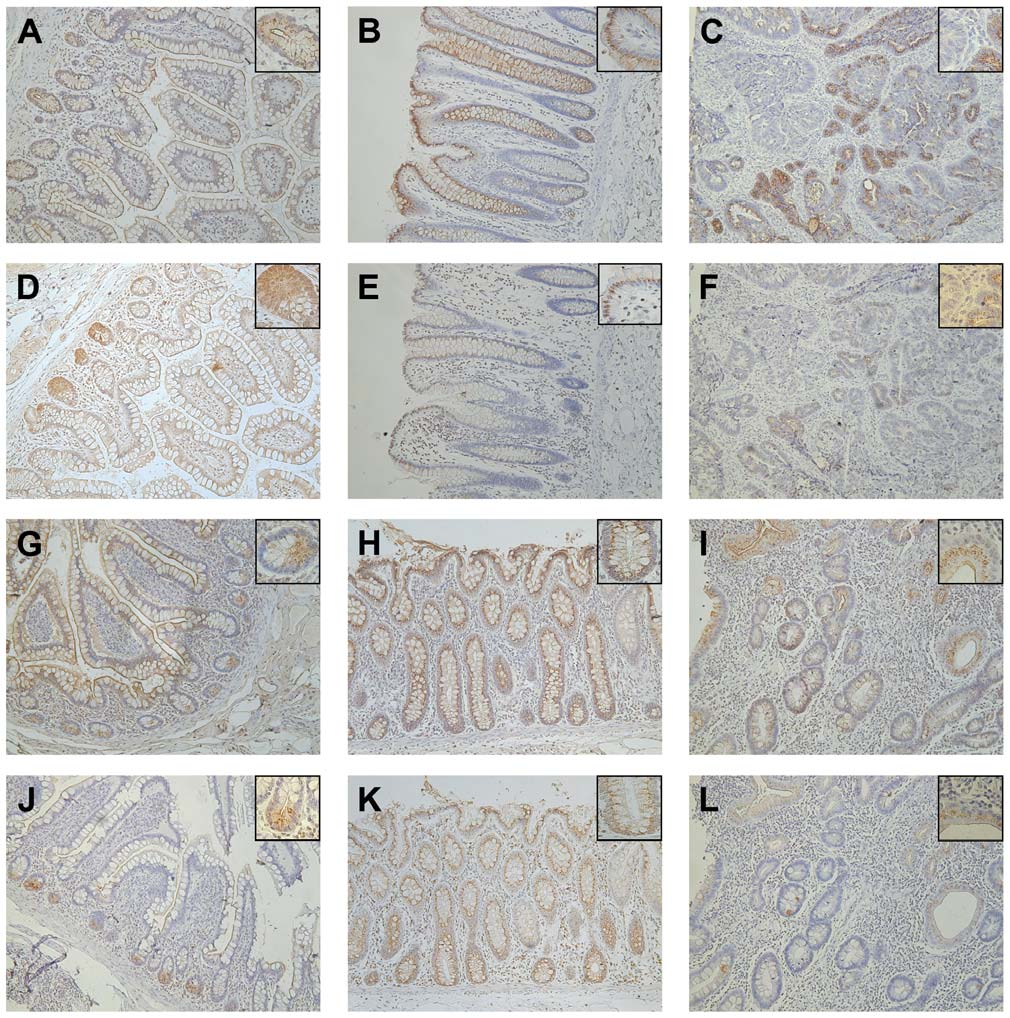
Positive staining for FcεRI α-chain and defensin-5 is observed in serial sections from intestinal tissue. (A) FcεRI α-chain is detected on the membrane, as well as in the cytoplasm of epithelial cells in small intestine of cancer patient No. 16. FcεRI α-chain positive cells are also found in (B) the colon and (C) a tumor sample from the same patient. (D) Staining with anti-defensin-5 antibodies confirmed that FcεRIα expressing cells at the basis of the small intestinal crypts are Paneth cells. Defensin-5 is expressed also in (E) colon and (F) tumor sample in same areas, but to a lesser extend when compared with FcεRI α-chain staining. Similar staining pattern are observed also for the CD patient No. 8, as FcεRI α-chain positive cells are detected in (G) the epithelium of small intestinal tissue, (H) colon and (I) lesional region. Defensin-5 positive cells are located at (J) the crypt basis of small intestine, (K) along the colon crypt, as well as in (L) the lesional region. Original magnification ×10, inset ×40.

Immunohistochemical (IHC) analysis revealed positive staining for FcεRI α-chain in the intestinal epithelium in eight of eleven (8/11) (73%) colon cancer patients and in 5/11 (45%) patients with gastrointestinal inflammation. In most cases, both small intestine and colon were positive for FcεRI α-chain. However, one patient per group (No. 24 and 12, respectively) was positive only in the colon, and two tumor patients (No. 21 and 26) expressed FcεRI α-chain only in the small intestinal epithelium. The four control samples from patients without gastrointestinal disorders were negative for FcεRI α-chain, suggesting that receptor expression might be observed only with gastrointestinal pathology (Table 1). Figure 1 shows IHC analysis of tissue sections from a representative colon cancer (A–F) and a gastrointestinal inflammation patient (G–L). In the specimen from the tumor patient (No. 16), the FcεRI α-chain was expressed in membrane and cytoplasm of the small intestinal epithelial cells throughout the entire intestinal villus and crypt. At the basal side of crypt cells, the positive staining was found also in the supranuclear region, most likely the Golgi apparatus (Figure 1A). Staining serial sections with the Paneth cell marker defensin-5 identified FcεRI α-chain positive cells in the small intestinal crypts as Paneth cells (Figure 1D). The observed staining in the patient with colon cancer was comparable to that seen in the small intestine of the CD patient (No. 8, Figure 1G, J). Interestingly, we observed a positive correlation between FcεRI staining in the epithelial cells of the ileum and colon: the majority of patients expressing the receptor in the small intestine (Figure 1A, G) had positive staining also in the colon (Figure 1B, H). In addition, we found FcεRI α-chain positive cells in the tumor samples of colon cancer patients (6/11) and in the lesion areas of three patients with gastrointestinal inflammations (3/11) (Table 1; Figure 1C, I).

**Table 1:** Patients' characteristics and staining results.

				Small intestine		Colon		Lesion/tumor	
Pat. No.	Sex	Age	Pathology (Tumor staging)	FcεRI-α	Def. 5	FcεRI-α	Def. 5	FcεRI-α	Def. 5
1	f	41	–(obese patient)	−	+	n.d.	n.d.	n.a.	n.a.
2	f	39	–(obese patient)	−	+	n.d.	n.d.	n.a.	n.a.
3	f	19	–(biopsy, diarrhea, meteorism)	−	+	−	−	n.a.	n.a.
4	f	50	–	−	+	n.d.	n.d.	n.a.	n.a.
5	m	47	Crohn's disease, active phase	+ Pc, M, sV	+	+	+	+	+
6	f	35	Crohn's disease, active phase	−	+	−	−	−	+
7	f	27	Crohn's disease, active phase	−	+	−	+	−	+
8	f	37	Crohn's disease, active phase	+ Pc, M, V	+	+ M	+	+	+
9	m	19	Crohn's disease, active phase	−	+	−	−	−	+
10	f	61	Crohn's disease, active phase	−	+	−	−	−	+
11	m	28	Crohn's disease, active phase	−	+	−	−	−	+
12	f	29	Crohn's disease, active phase	−	+	+ M	−	−	+
13	m	50	Diverticulitis and Peridiverticulitis	−	+	−	−	−	+
14	f	82	Inflammation	+ Pc, M, V	+	+ M	+	+	+
15	f	30	C-Gastritis (biopsy)	+ Pc	+	n.a.	n.a.	n.a.	n.a.
16	m	66	Invasive, moderately well differentiated adenocarcinoma, ascending colon (G2, pT3, pN2, pM1, Dukes D)	+ Pc M, V	+	+	+	+	+
17	f	78	Invasive, moderately well differentiated adenocarcinoma, ascending colon (G2, pT2, pN1, Dukes C1)	−	+	−	−	−	−
18	f	69	Invasive, moderately well/moderately differentiated adenocarcinoma, Cecum (G2/G3, pT3, pN1, Dukes C)	−	−	−	−	−	+
19	f	67	Invasive, low differentiated adenocarcinoma, ascending colon (G3, pT3, pN0, pM1)	+ Pc	+	+	−	+	−
20	m	76	Invasive, low differentiated adenocarcinoma, ascending colon (G3, pT3, pN0, pMX, V1)	+ Pc, M, V	+	+	−	−	−
21	m	84	Invasive, well differentiated adenocarcinoma, ascending colon (G1, pT2, pN0, pMX, Dukes B)	+ Pc, sV	+	−	+	+	−
22	m	70	Invasive, moderately well/moderately differentiated adenocarcinoma, ascending colon (G3, pT3, pN2, pMX, Dukes C1)	−	+	−	−	−	−
23	m	68	Invasive, moderately well differentiated adenocarcinoma, Cecum (G2, pT3, pN0, pMX, Dukes B)	+ Pc, M	−	+	−	+	**−**
24	m	57	Invasive, moderately well differentiated adenocarcinoma, Cecum (G2, pT4, pN0, V1, Dukes B)	−	+	+	+	−	−
25	m	79	Invasive, moderately well differentiated adenocarcinoma, ascending colon (pT3, pN0, pMX, Dukes B)	+ Pc, M	+	+	−	+	−
26	f	86	Invasive, moderately well differentiated adenocarcinoma, ascending colon (pT4, pN2, pM1, V1, L1, Dukes D)	+ Pc, V	+	+	+	+	+

n.d., not determined; n.a., not applicable; Pc, Paneth cell staining; M, Membrane staining; sV, single cell staining in villi; V, all villus epithelium.

Surface membrane localization of FcεRI α-chain on small intestinal epithelial cells was observed in tissue samples from 4/11 of cancer patients and in 4/11 of patients with gastrointestinal inflammations (one small intestine, two small intestine + colon, one colon). The IHC analysis as indicated in Table 1 was validated by immunofluorescence (IF) staining.

**Figure 2:**
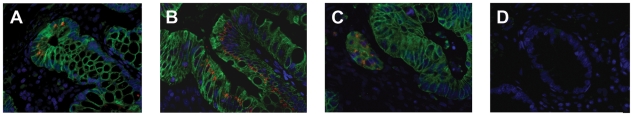
FcεRI α-chain is expressed in epithelial cells. Co-staining with anti- FcεRI α-chain (red) and anti-keratin 8 antibodies (green) verifies the epithelial expression of FcεRI in (A) the crypts of small intestinal section, where FcεRIα is found primarily in the supranuclear region, in (B) colon tissue and in (C) tumor sample of cancer patient No. 16. (D) Negative control with mouse IgG2b and rabbit IgG. The blue fluorescence DAPI staining indicates the nuclei. Original magnification ×40.

We further confirmed that FcεRI α-chain is expressed in small intestinal, colonic and lesion/tumor epithelial cells by IF double-staining with the epithelial cell marker cytokeratin 8 (Figure 2).

**Figure 3:**
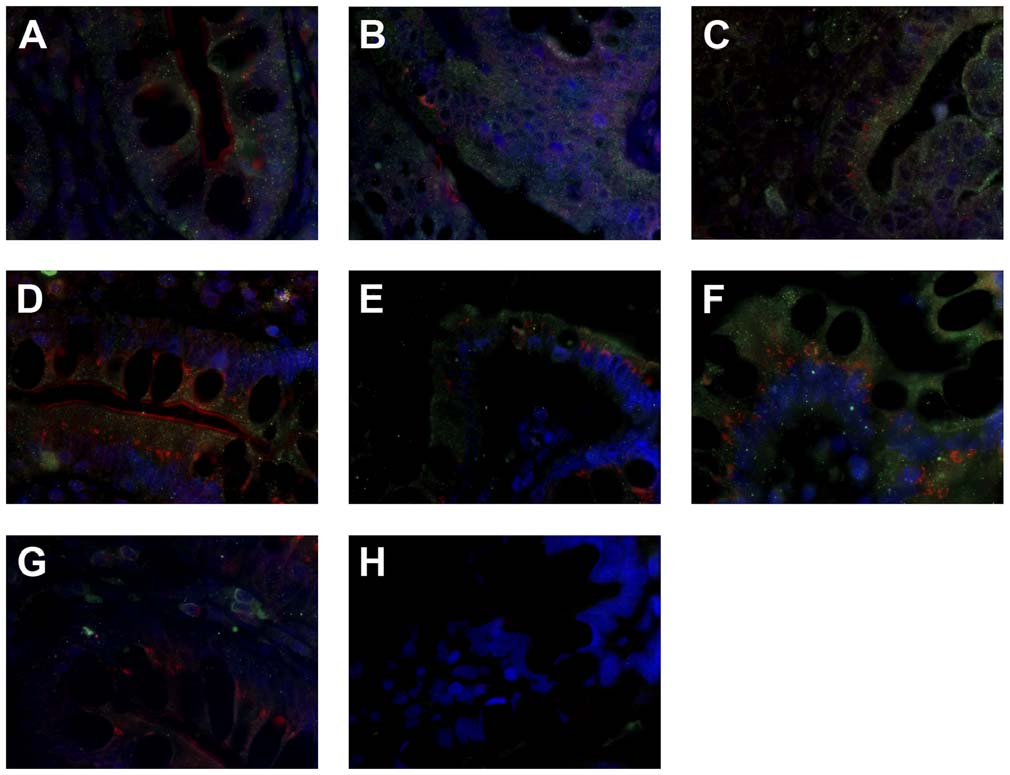
Co-expression of FcεRI α- and γ-chain, but not FcεRI α- and β-chain is observed in epithelial cells of intestinal tissue. In sections from cancer patient No. 23 FcεRI γ-chain (green) is found to be co-expressed in FcεRIα (red) positive epithelial cells of (A) the small intestine, as well as in (B) colon tissue and (C) tumor sample. In addition, sections from CD patient No. 8 are double-positive for FcεRI α- and γ-chain in (D) the small intestinal, (E) colon and in (F) lesional tissue. (G) Only in the subepithelial tissue FcεRI β-chain (green) positive cells are detected as shown here in the crypts of small intestinal tissue. (H) Negative control with mouse IgG2b and goat IgG isotype control antibodies. The blue fluorescence DAPI staining indicates the nuclei. Original magnification ×64.

In addition to the α-chain subunit, a functional FcεRI complex requires the presence of a γ-chain subunit, with or without a β-subunit. Therefore, we conducted IF analysis to determine whether the FcεRIα-chain was co-expressed with either the β- or γ-chains. These experiments revealed a double-staining for α- and γ-chain along or at the top of the villi in all seven FcεRIα positive small intestinal tissue samples from tumor patients, as well as in 3/4 FcεRIα positive small intestinal tissue samples from patients with gastrointestinal inflammation (Table 2; Figure 3A, D). In addition to the small intestine, co-expression of the α- and γ-chain was observed in 4/6 FcεRIα positive colon samples from tumor patients and in 2/3 colon sections from gastrointestinal inflammation patients (Table 2; Figure 3B, E). A correlation of γ-chain with α-chain co-expression was found in 4/6 FcεRIα positive tumor samples as well as in all lesional tissues (Table 2; Figure 3C, F). Nevertheless, no positive staining for the FcεRI γ-chain was observed in Paneth cells at the base of the crypts (data not shown). In contrast to α- and γ-chain co-expression, the β-chain of FcεRI could be detected, with one exception, only in cells of the subepithelial stroma, but not in the intestinal epithelial layer (Table 2; Figure 3G). The exception was colonic epithelial cells in tumor patient No. 25 (Table 2), where FcεRI β-chain was detected exclusively in the supranuclear region, most likely the Golgi apparatus.

**Table 2:** FcεRI β- and γ-chain staining results in FcεRI α-chain expressing intestinal tissue.

	Small intestine			Colon			Lesion/tumor		
Pat. No.	FcεRI-α	FcεRI-β	FcεRI-γ	FcεRI-α	FcεRI-β	FcεRI-γ	FcεRI-α	FcεRI-β	FcεRI-γ
5	+	−	+	+	−	+	+	−	+
8	+	−	+	+	−	+	+	−	+
14	+	−	+	+	−	−	+	−	+
15	+	−	−	n.a.	n.a.	n.a.	n.a.	n.a.	n.a.
16	+	−	+	+	−	+	+	−	+
19	+	−	+	+	−	−	+	−	−
20	+	−	+	+	−	−	−	−	−
21	+	−	+	−	−	−	+	−	−
23	+	−	+	+	−	+	+	−	+
25	+	−	+	+	+	+	+	−	+
26	+	−	+	+	−	+	+	−	+

n.a., not applicable;

Analysis of various clinical parameters, including patients' age, sex and pathological disease classification showed no association with staining results for FcεRI expression.

### Epithelial FcεRI Exhibits IgE Binding Properties

**Figure 4:**
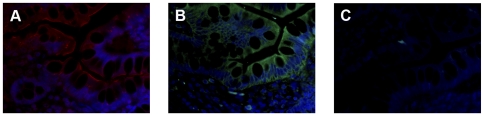
FcεRI positive tissue reveals IgE binding activity. Immunofluorescence staining of serial sections from patient No. 8 revealed IgE binding in (A) FcεRI α-chain (red) positive epithelial cells being incubated with (B) monoclonal NiP-specific humanized IgE antibodies (green). (C) Negative control with PBS. The blue fluorescence DAPI staining indicates the nuclei. Original magnification ×40.

To determine whether the FcεRIαγ_2_ expressed in the intestinal epithelium had specific antibody binding capacity, we performed IgE binding experiments on paraffin-embedded intestinal tissue sections. Samples positive for FcεRIα on the apical cellular membrane (Figure 4A) showed specific binding of human serum IgE or humanized anti-4-hydroxy-3-nitrophenylacetyl (NiP)-specific IgE (Figure 4B), with indistinguishable binding patterns. No epithelial binding was seen with unspecific human IgG antibodies or FITC-labeled dextran used as controls (data not shown). The observed IgE-FcεRI interactions correlated with the FcεRI α-chain expression in the epithelium, being expressed on the epithelial membrane as well as in the cytoplasm (Figure 4A, B). The IgE binding was not inhibited by anti-CD23 preincubation or by lactose, indicating protein- rather than carbohydrate-mediated IgE binding.

### High Levels of FcεRI α- and γ-Chain mRNA Expression Are Detected in Intestinal Epithelial Cell Lines

**Figure 5:**
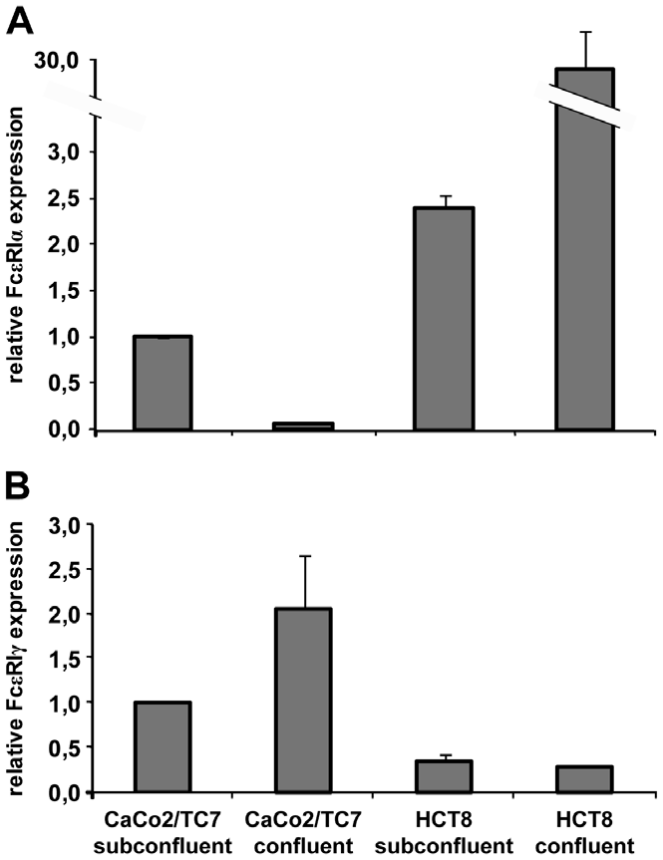
Expression of FcεRI α- and γ-chain but not β-chain mRNA in human intestinal epithelial cells. The expression pattern of the FcεRI complex was analyzed by real-time PCR analysis using specific primers for detection of (A) FcεRI α-, β- (not shown), and (B) γ-chain. Target gene expression levels were normalized to the average of housekeeping genes and are depicted relative to the value of subconfluent Caco-2/TC7 cells. The values are presented as means +/− SD (n = 3) from one experiment. The results are representative of two independent experiments.

To analyze the expression of the FcεRI complex on the transcriptional level in human intestinal epithelial cells, total RNA was isolated from subconfluent and confluent Caco-2/TC7 and HCT-8 cell lines. Human HMC-1 mast cells and transfected RBL-SX38 cells were used as positive controls, because they are known to abundantly express FcεRI α-, β- and γ-chains. Real-time PCR analysis revealed the presence of the FcεRI α-chain mRNA in all four intestinal cell lines, with highest expression in confluent HCT-8 cells (Figure 5A). The expression level was approximately half that observed in the HMC-1 positive control cells (data not shown). The γ-chain mRNA also was found in all four intestinal cell samples, with highest levels detected in confluent Caco-2/TC7 cells (Figure 5B). In this case, the mRNA expression level of γ-chain was 80-times higher in the HMC-1 cells (data not shown). None of these intestinal epithelial cell lines expressed detectable FcεRI β-chain mRNA (data not shown). In the second positive control cell line, high mRNA levels of all three FcεRI chains were observed, reflecting that these transfected RBL-SX38 cells over-express FcεRI (data not shown). Together, these findings verify the presence of FcεRI α- and γ-chain mRNA, the components of the trimeric FcεRIαγ_2_ complex, in human intestinal epithelial cells.

### Highest Expression of FcεRI α- and γ-Chains Is Observed in Undifferentiated, Subconfluent Intestinal Cell Lines

**Figure 6:**
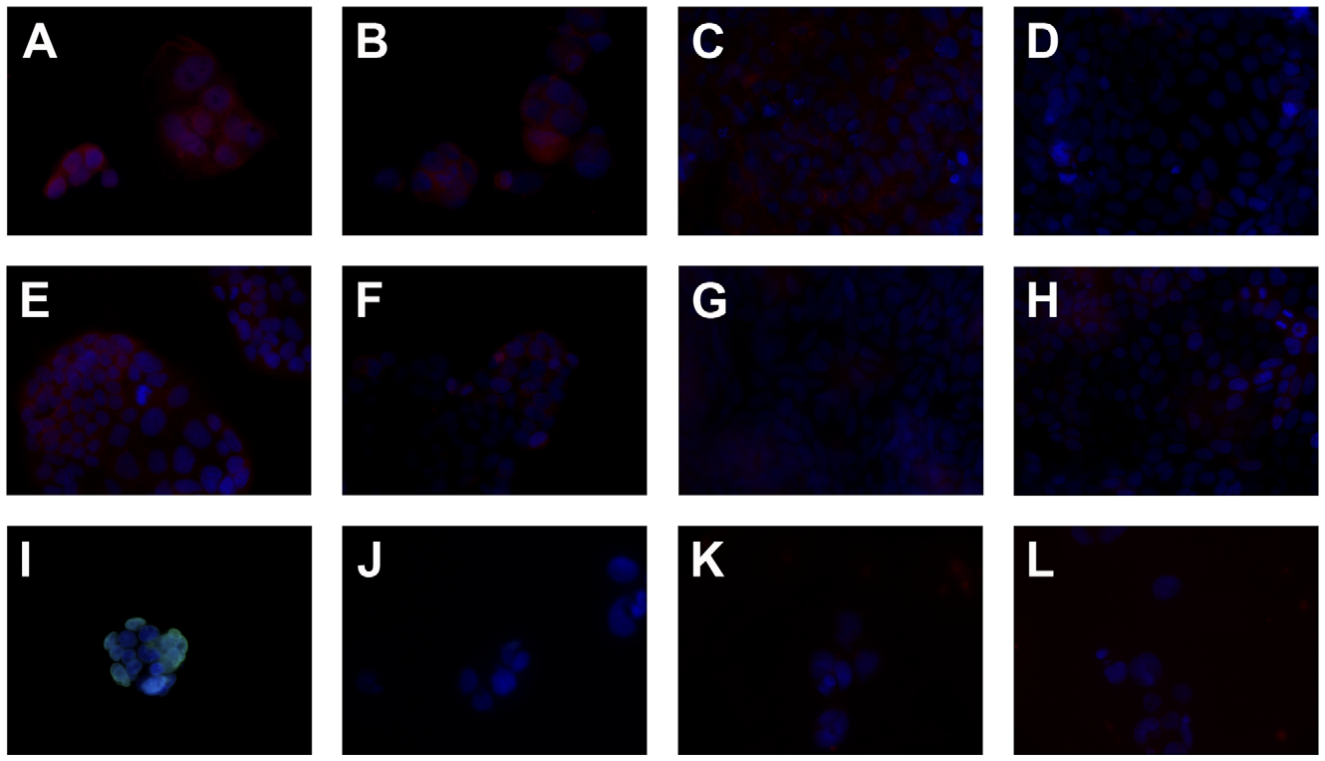
Abundant surface and cytoplasmatic FcεRI α-chain expression only in subconfluent human intestinal tumor cell lines. Immunofluoresence staining for (A–H) FcεRIα is performed in (A, B) subconfluent and (C, D) confluent Caco2/TC7 as well as in (E, F) subconfluent and (G, H) confluent HCT-8. Triton-X-100 permeabilized (A, C, E, G) and untreated cells (B, D, F, H) were compared. (I–L) Representative control staining in subconfluent Caco2/TC7 with the (I, J) anti-lamin A/C or (K, L) unspecific murine IgG2b, (I, K) permeabilized or (J, L) untreated. The blue fluorescence DAPI staining indicates the nuclei. Original magnification ×40.

To screen intestinal epithelial cell lines for FcεRI α-, β- and γ-chain protein expression we performed IF staining. We observed abundant α-chain expression in undifferentiated, subconfluent Caco-2/TC7 (Figure 6A, B) and HCT-8 cells (Figure 6E, F). To exclude intracellular staining signals for FcεRIα, we confirmed membrane integrity by comparing Triton-X-100 permeabilized cells with untreated controls using lamin A/C staining (Figure 6I, J). The total (Figure 6A) and surface-membrane expression (Figure 6B) were comparable in Caco-2/TC7 cells, whereas in subconfluent HCT-8, the surface expression of FcεRIα was less pronounced (Figure 6F) compared to the staining observed in the permeabilized cells (Figure 6E). The expression levels decreased with confluency, as only a faint staining could be detected in the cytoplasma of confluent Caco-2/TC7 (Figure 6C) and HCT-8 cells (Figure 6G). No FcεRIα positive staining was observed on the membrane of confluent Caco-2/TC7 cells (Figure 6D). In the HCT-8 cells grown as multilayers with the new cells revealing a more immature phenotype, a scarce positivity was seen (Figure 6H).

**Figure 7:**
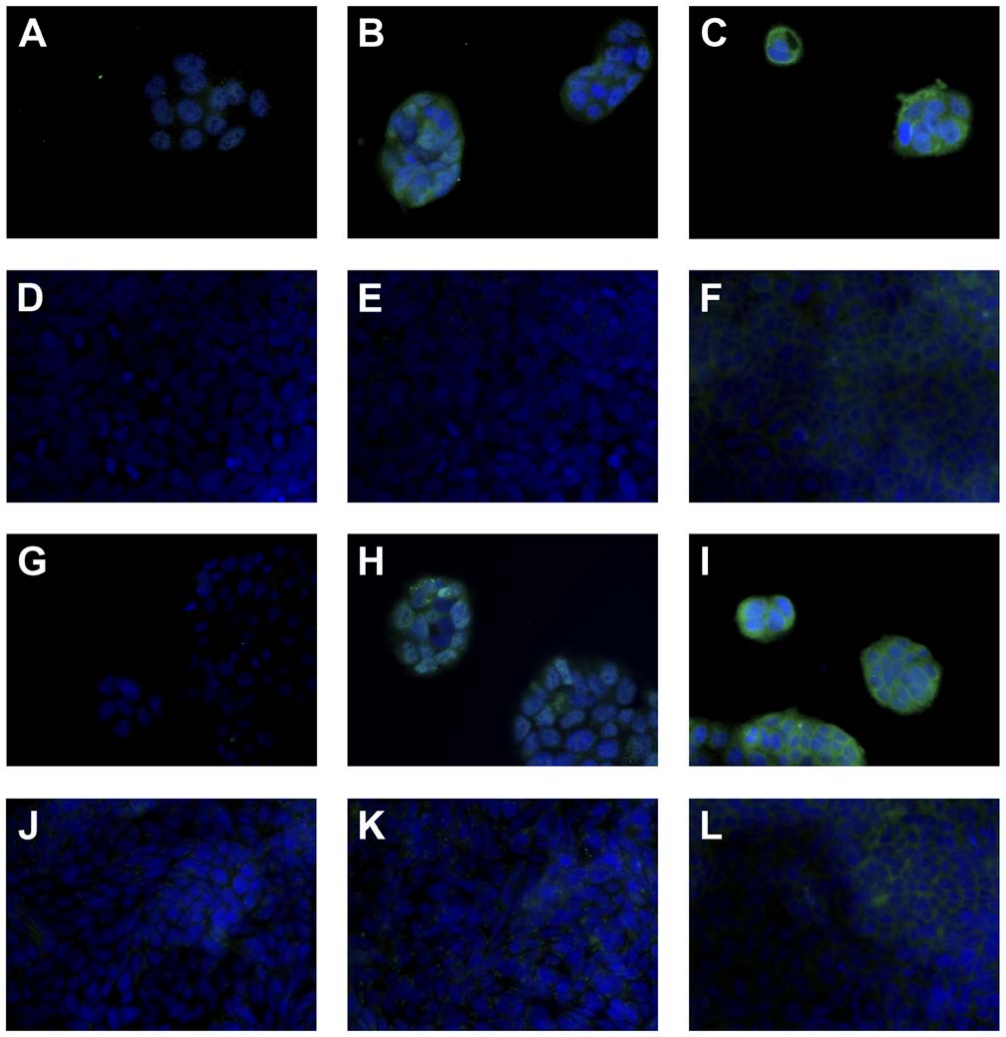
Abundant FcεRI γ-chain expression and IgE binding in subconfluent human intestinal tumor cell lines. Immunofluorescence staining of (A, D, G, J) FcεRI β- and (B, E, H, K) FcεRI γ-chain are performed in (A–C) subconfluent and (D–F) confluent Caco2/TC7 and in (G–I) subconfluent and (J–L) confluent HCT-8. (C, F, I, L) According to the expression pattern of FcεRI in the subconfluent cells, IgE binding is observed exclusively in subconfluent, non-mature intestinal cells. The blue fluorescence DAPI staining indicates the nuclei. Original magnification ×40.

We could not detect FcεRIβ in any intestinal epithelial cell line irrespective of the state of confluence (Figure 7A, D, G, J). In contrast, a prominent FcεRI γ-chain expression was seen in subconfluent Caco-2/TC7 (Figure 7B) and HCT-8 cells (Figure 7H), but not after confluence (Figure 7E, K). Consistent with these results, binding of IgE was high in the subconfluent Caco-2/TC7 (Figure 7C) and HCT-8 cells (Figure 7I), whereas only a background staining was seen in confluent Caco-2/TC7 (Figure 7F) and HCT-8 (Figure 7L) cells. The IgE binding was not inhibited by anti-CD23 preincubation.

**Figure 8:**
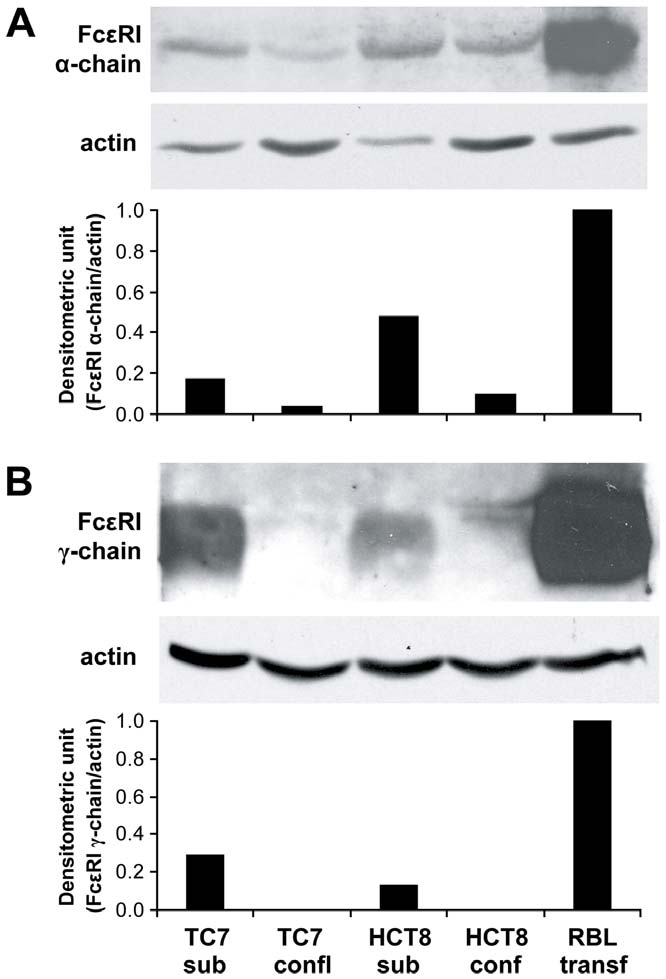
Western blot analysis reveals FcεRI in human intestinal tumor cell lines. (A) FcεRI α-chain and (B) FcεRI γ-chain expression is investigated in Caco2/TC7 and HCT8 subconfluent and confluent cell lines. In all experiments RBL cells transfected with the human FcεRI receptor served as positive controls and the protein expression signal was normalized to the expression of house-keeping protein actin.

The IF staining of Caco-2/TC7 and HCT-8 cells was confirmed by western blot analysis with cell lysates obtained from subconfluent and confluent cells. We observed a 45-kDa protein band of FcεRI α-chain in all cell lysates, with a stronger signal in the subconfluent cells (Figure 8A). The band was rather sharp, even though FcεRI α-chain is known to be glycosylated. In contrast, we could detect the 10 kDa protein band of FcεRI γ-chain only in subconfluent Caco-2/TC7 and HCT-8 cells (Figure 8B). Both the α- and γ-chains were detected in the positive control RBL cell line over-expressing the human FcεRI (Figure 8B). High amounts of expressed FcεRI α- and γ-chain proteins were seen in the human mast cell line HMC-1 (data not shown). Thus, our data indicate that a functional high affinity IgE receptor is expressed by these cell lines when they are subconfluent and not after confluence.

## Discussion

In the present study, we investigated the expression of FcεRI in the human intestinal epithelium and analyzed the role of cell growth or confluency on receptor expression in intestinal epithelial cell lines. We observed pronounced FcεRI α-chain expression in 73% of colon cancer patients and in 45% with gastrointestinal inflammations. *In vitro* experiments revealed only undifferentiated, subconfluent intestinal epithelial cells to express the α- and γ-chains of FcεRI. The functionality of the receptor was evident by the specific binding of IgE to the FcεRI α-chain.

It is easy to speculate that IgE antibodies might contribute to gastrointestinal inflammatory disorders [4]–[6]. The expression of the IgE high affinity receptor in cancer patients could have beneficial or detrimental effects. On the one hand, IgE antibodies have been suggested to participate in tumor immunosurveillance [9]. However, other facts could argue for a role of FcεRI in tumorigenesis. IgE binding to FcεRI was previously reported to both stabilize the receptors on the surface leading to receptor accumulation [32], but also to enhance survival of FcεRI bearing mast cells by autocrine cytokine secretion and induction of an anti-apoptotic protein [33]. Additionally, FcεRI signaling induces Ras activity, which in mast cells is associated with cell growth, differentiation and survival. Similarly, Ras initiates neoplastic cells proliferation via the inositol (1,4,5) triphosphate 3-kinase and ERK signaling [34], [35]. Therefore, our data on FcεRI expression in colon tumor specimens, as well as in subconfluent, proliferating cell lines, might reflect a role for this receptor in tumor cell growth and survival.

It is well known that intestinal epithelial cells express HLA class II antigens on their surface and therefore are able to function as antigen presenting cells [36], [37]. Interestingly, the cross-linking of trimeric FcεRIαγ_2_ expressed on professional antigen presenting cells [12] by IgE and bound antigen was shown to induce endocytosis of the complex and uptake into MHC class II-rich compartments [16]. In this context, in IgE mediated disorders such as allergy a more enhanced efficiency of antigen-presentation to T cells was reported [38], [39]. Thus, expression of FcεRI might contribute to the antigen presenting function of intestinal epithelial cells.

It is also possible that intestinal epithelial expression of FcεRI could exert a shuttle function for specific IgE through the intestinal epithelium. In diseases with elevated levels of IgE antibodies an increased intestinal transepithelial transport of IgE antibodies has been demonstrated, resulting in detectable amounts of IgE antibodies in feces [40]. From animal experiments, it is known that this IgE transport to the lumen occurs through intact epithelial barrier [41]. To date, only scant information is available regarding the mechanisms of this transepithelial IgE transport, even though antibody-binding structures seem to play a defined role. So far, only CD23 has been implicated in enhanced IL-4 dependent antibody shuttling through the intestinal epithelium [22], [23]. The same IgE/CD23 shuttle mechanism seems to be responsible for transepithelial food allergen uptake and protect the antigens from degradation during this transport [24]. Based on the data presented here, FcεRI on enterocytes could contribute to IgE transfer through the gut epithelium. As no inhibition of IgE binding was seen in our experiments using anti-CD23 antibodies, the higher affinity of FcεRI to the Fc domain of IgE [10] could possibly enhance IgE transport.

Upon screening of the intestinal epithelium for FcεRI we found FcεRI α- but not γ-chain expression at the base of the crypts at the level of Paneth cells, as suggested by previous work of our group [42]. Interestingly, intracellular expression and cellular release of FcεRIα was reported for other cell types such as eosinophils [43] and soluble IgE receptors were found to exert distinct biological functions such as regulation of IgE production, T cell and granulocyte maturation and macrophage migration [44]. Thus, Paneth cells might be of special interest as they contribute substantially to innate immunity in the intestine [45]. Further, Paneth cells appear to have a central role in some forms of inflammatory bowel disease [46]. Paneth cells are primarily present in small intestinal tissue in healthy individuals. However, metaplastic defensin-5 positive cells were found in the upper gastrointestinal tract and the diseased colon, and are considered an early marker of epithelial dysplasia and cancer development [47], [48]. In contrast to the absorptive epithelial cells, which migrate from the crypts to the top of the villi during their 4–6 days lifespan [49], Paneth cells are found adjacent to the rapidly dividing pluripotent stem cells. Thus, alternative maturation signals are crucial for Paneth cell differentiation [50]. Even though real-time PCR analysis revealed the presence of the FcεRI α- and γ-chains mRNA in confluent intestinal cells, only undifferentiated proliferating cells expressed a functional FcεRIαγ_2_ as detected by cellular IF staining and Western blot experiments. This expression was decreased in confluent, differentiated cells. Thus, protein expression of FcεRI α- and γ-chain in intestinal epithelial cells might be mainly regulated on the translational level.

Our data demonstrate that the FcεRI α- and γ-chains, which are crucial components of the heterotrimeric FcεRIαγ_2_ isotype, are expressed in intestinal epithelial cells of patients with colon cancer or gastrointestinal inflammation. Although these data do not elucidate the precise function of FcεRI in the intestinal epithelium, the novel findings suggest that FcεRI may contribute to immunosurveillance or pathophysiology at the intestinal mucosa.

## Materials and Methods

### Patient Samples

Four-micron sections of formalin fixed (buffered formalin, pH 7.5) paraffin-embedded tissue of small intestinal and colon specimens were investigated. The samples were obtained from eleven patients with gastrointestinal inflammations and eleven patients with colon cancer. In each case, mucosa with pathological changes as well as regular mucosa without any histological signs of abnormality was investigated. Additionally, sections of biopsies from four patients without gastrointestinal diseases served for control purposes. Patient characteristics are summarized in Table 1. The inclusion of human specimens for the present study was approved by the ethic's committee of the Medical University Vienna (number: 317/2007). The protocol permitted only usage of numerically coded human tissue sections from therapeutical interventions. The authors of the study had no access to patients' data except the information given in Table 1. Thus, the ethic's committee of the Medical University Vienna waived informed consent.

### Antibodies

We used the following anti-human primary antibodies in this study: mouse monoclonal anti-FcεRI α-chain (clone CRA1) purchased from Cosmo Bio (Co., Tokyo, Japan), rabbit polyclonal anti-FcεRI α-chain purchased from Upstate (Lake Placid, NY), mouse monoclonal anti-defensin-5 (clone 1G11) generated by C. L. Bevins [47], goat polyclonal anti-FcεRI β-chain, goat polyclonal anti-FcεRI γ-chain, mouse monoclonal anti-CD23 (clone BU38) and goat polyclonal anti-lamin A/C purchased from Santa Cruz Biotechnology Inc. (Santa Cruz CA), rabbit polyclonal anti-FcεRI γ-chain purchased from Abcam (Cambridge, UK), rabbit anti-keratin 8 purchased from NeoMarkers Inc (Fremont, CA), mouse monoclonal anti-actin purchased from Chemicon International, (Millipore, Billerica, MA); human serum IgE purchased from Alpha Diagnostic Intl. Inc. (San Antonio TX), humanized murine anti-NiP IgE antibody purchased from Serotec (Oxford, UK). Non-specific mouse IgG1 and IgG2b (Jackson ImmunoResearch Laboratories, West Grove, PA), non-specific goat IgG (Zymed Laboratories San Francisco, CA) and non-specific rabbit IgG (Serotec, Oxford, England) served as negative controls. Specific binding of antibodies was detected using the following secondary antibodies or detection reagents: goat anti-mouse AlexaFluor 568-conjugated and goat anti-rabbit AlexaFluor 488-conjugated purchased from Molecular Probes (Invitrogen, Carlsbad, CA), biotinylated rabbit anti-goat and FITC-Streptavidin purchased from Dako (Carpinteria, CA), FITC-labeled donkey anti-goat (Jackson ImmunoResearch Laboratories), FITC-labeled goat anti-human IgE purchased from Vector Laboratories (Burlingame, CA); HRP-conjugated anti-rabbit purchased from Amersham Biosciences (GE Healthcare, UK), HRP-conjugated anti-mouse purchased from Jackson Immunoresearch Laboratories. 4-hydroxy-3-nitrophenylacetyl coupled in a ratio of 15∶1 to bovine serum albumin (NiP-BSA) was purchased from Biosearch Technologies (Novato, CA). FITC labeling of NiP-BSA was performed with the EZ-Label FITC protein labeling kit (Pierce, Rockford, IL) following the manufacturer's instructions.

### Immunohistochemical Staining of Intestinal Tissue Sections

The IHC staining procedure is described elsewhere [51]. Briefly, sections were deparaffinized and rehydrated. After antigen retrieval with 10 mM citrate buffer pH 6, the endogenous peroxidase activity was quenched by exposure to 3% H_2_O_2_ in methanol. Sections were permeabilized with PBS/0.2% Tween, and unspecific antibody binding was blocked with 5% horse serum. The primary antibodies used were mouse monoclonal anti-FcεRI α-chain (Cosmo Bio), and mouse monoclonal anti-defensin-5 (clone 1G11) [47]. Murine IgG1 and IgG2b served as negative controls. After washing, the sections were further processed with a Vectastain Elite ABC kit (Vector Laboratories, Burlingame, CA) following the manufacturer's instructions. Color reaction was developed with a DAB^+^ chromogen kit (Dako). After counterstaining with hematoxylin the sections were dehydrated and mounted with Eukitt, and then analyzed using a Nikon Eclipse E400 microscope (Nikon, Vienna, Austria).

### Immunofluorescent Double-Staining

Sections were treated as described above with slight modifications. After antigen unmasking with 10 mM citrate buffer pH 6 and permeabilization with PBS/0.2% Tween, the sections were blocked with 5% goat serum. For the FcεRI α-chain/cytokeratin 8 double staining, the first primary antibody applied was mouse anti-FcεRI α-chain (Cosmo Bio) overnight at 4°C. Mouse IgG2b served as negative control. After washing goat anti-mouse AlexaFluor 568 was applied for 1 h. After washing and blocking again with 5% goat serum, the second staining was performed using rabbit anti-keratin 8 as the second primary antibody (or rabbit IgG as negative control) for 1 h. After washing, goat anti-rabbit AlexaFluor 488 secondary antibody was applied for 1 h. For the double-staining of FcεRI α-chain with FcεRI β- or γ-chain the first primary antibody applied was goat anti-FcεRI β- or γ-chain overnight at 4°C after blocking with 5% rabbit serum. Goat IgG was used as negative control. After washing the sections were incubated with biotinylated polyclonal rabbit anti-goat Ig for 30 min at 37°C. The sections were again washed and incubated with FITC-Streptavidin for 30 min at room temperature. After washing and blocking again with 5% goat serum, the second staining was performed using the mouse anti-FcεRI α-chain as the second primary antibody (or mouse IgG2b as negative control) for 1 h. After washing goat anti-mouse AlexaFluor 568 was applied for 1 h. The sections were washed and counterstained with 4′,6-Diamidino-2-phenylindole 0.1 µg/ml (Molecular Probes). Afterwards, sections were mounted in Vectashield medium (Vector Laboratories) and investigated in a Zeiss Axioplan 2 microscope (Carl Zeiss Göttingen, Germany).

### Human IgE Binding in Intestinal Tissue Sections

For IgE binding experiments, human intestinal sections were cleared in xylene, rehydrated and subjected to heat-mediated antigen retrieval as described above. Residing antibodies were stripped by low pH treatment, and unspecific binding sites were blocked with 5% BSA/PBS for 30 min. To block IgE binding to Galectin and CD23, α-Lactose 25 mM and anti-CD23 (clone BU38) were applied overnight at 4°C. Thereafter, slides were incubated with 10 µg/ml NiP-IgE, serum IgE or PBS for 2 h at 37°C. For controls, either 5 µg/ml FITC-labeled IgG or dextran in PBS/1% BSA were incubated overnight at 4°C under light protection. The sections were then incubated with either FITC-labeled NiP-BSA (NiP-IgE) or goat anti-human IgE (NiP-IgE and serum IgE). The sections were further processed for IF or IHC analysis as described above. Only the results generated with NiP-IgE/FITC-labeled anti-human IgE are shown.

### Cell Culture

All cell culture media were supplemented with 10% heat inactivated FCS (PAA Laboratories, Pasching, Austria), 4 mM glutamine, 100 IU/ml penicillin and 100 µg/ml streptomycin if not explicitly stated. The human adenocarcinoma cell line HCT-8 (originating from the ileocecal region) (kindly provided by Gerhard Hamilton, Department of Surgery, Medical University Vienna, Vienna, Austria) was grown in RPMI 1640 supplemented with 10% FCS (PAA Laboratories, Pasching, Austria) and 4 mM glutamine. The human colon adenocarcinoma cell line Caco-2 clone TC7 (Caco-2/TC7) (kindly provided by Monique Rousset, INSERM, Paris, France) was grown in Dulbecco modified minimal essential medium + GlutaMAX-II with high glucose (4500 mg/ml), supplemented with 1% non-essential aminoacid, 10 mM HEPES. Cells were used at subconfluent stage (60% confluence) or 10 days after confluency. For all experiments RBL-SX38 cells which are transfected with the complete human FcεRI (kindly provided by Jean-Pierre Kinet, Harvard Institute of Medicine, Boston, MA) and human HMC-1 mast cells (kindly provided by Joseph H. Butterfield, Mayo Clinic, Rochester, MN) served as a positive control. RBL-SX38 cells were cultured in RPMI 1640 medium, HMC-1 cells were grown in IMDM.

### RNA Isolation, Reverse Transcription and Real-Time PCR Analysis

Total RNA was isolated from subconfluent and confluent human Caco-2/TC7 and HCT-8 intestinal cell lines by using the RNeasy RNA isolation Kit (Qiagen, Vienna, Austria), including on-column DNase digest following the manufacturer's instruction. RNA integrity was analyzed by agarose gel electrophoresis. RNA concentration and purity was determined using a Biophotometer (Eppendorf, Hamburg, Germany). RNA isolated from HMC-1 and RBL-SX38 cells was used as a positive control for evaluation of target gene (human FcεRI α-, β-, and γ-chain) expression. Two µg of total RNA were transcribed into cDNA using the High Capacity cDNA Reverse Transcription Kit (Applied Biosystems, Vienna, Austria).

Expression analysis was performed by real-time PCR on a StepOne Plus real-time PCR system (Applied Biosystems). The analysis was carried out with a two step protocol starting with 20 seconds at 95°C, followed 40 cycles of 1 second at 95°C and subsequent 20 seconds at 60°C. In all experiments triplicates were set up containing the POWER SYBR Green PCR Master Mix (Applied Biosystems) reagent. Primers were designed using “Primer Express 2.x” software (Applied Biosystems) and span, when possible, exon–intron boundaries to avoid signals from contaminating genomic DNA. The forward (fwd) and reverse (rev) primers consist of the following sequences: beta Actin fwd: tggctcccgaggagcac, rev: ccttaggtttctgagggactgcta; RPLP0 fwd: cctcatatccgggggaatgtg, rev: gcagcagctggcaccttattg; FcεRIα fwd: catggaatcccctactctactgtgt, rev: ccttaggtttctgagggactgcta; FcεRIβ fwd: gagaaatgcaacatatctggtgagag, rev: aggttgatgatcaggatggtaattc; FcεRIγ fwd: caagtgcgaaaggcagctataa, rev: tgctcaggcccgtgtaaac.

Based on melting curve analysis no primer–dimers were generated during the PCR amplification. For relative quantification, data were analyzed by ΔΔCT method using “StepOne software 2.0” (Applied Biosystems). Expression levels of target genes were normalized to the average of house keeping genes, beta-actin and RPLP0, and are shown relative to the value of subconfluent Caco-2/TC7.

### Cell Immunostaining

Cells grown on glass cover slips were fixed for 20 min in 3% paraformaldehyde. After washing, the cells were incubated for 15 min with 50 mM NH_4_Cl to reduce spontaneous fluorescence. Unspecific binding sites were subsequently blocked by incubating cells with 5% goat serum. Cells were treated with mouse monoclonal anti-FcεRI α-chain (clone CRA1), goat polyclonal anti-FcεRI β-chain or goat polyclonal anti-FcεRI γ-chain (Santa Cruz Biotechnology Inc.) overnight at 4°C. Unspecific Mouse IgG2b or goat IgG were used as negative control. After washing the cells incubated with anti-FcεRI α-chain were treated with goat anti-mouse AlexaFluor 568 for 1 h. The cells incubated with anti-FcεRI β-chain or anti-FcεRI γ-chain were treated with biotinylated polyclonal rabbit anti-goat Ig for 30 min at 37°C followed by FITC-Streptavidin for 30 min at room temperature. For the cellular IgE binding assay a blocking step with anti-CD23 antibodies (clone BU38) applied overnight at 4°C was followed by incubation with 10 µg/ml NiP-IgE, serum IgE or PBS for 2 h at 37°C. The cells were then incubated with either FITC labeled NiP-BSA (NiP-IgE) or anti-human IgE antibodies (NiP-IgE and serum IgE). The cells were further processed for immunofluorescence analysis as described above. Only the experiments conducted with NiP-IgE/FITC labeled anti-human IgE are shown.

To analyze for membranous or total expression of FcεRI α-chain, cells were either permeabilized with 0.2% Triton-X-100 for 30 min, or left with PBS. Goat anti-lamin A/C antibody was used as positive control.

### Cell lysate and Western Blotting Analysis

Total protein cell extract and western blot analysis was performed as described before [52]. Briefly, after washing cells were homogenized in 1 ml lysis buffer (10 mM TRIS pH 7.4 containing 1% SDS). The lysate was boiled for 5 min, centrifuged at 1000 g, and then stored at −80°C. Equal amounts of protein were separated by 12% SDS-PAGE and blotted to a nitrocellulose membrane. Nonspecific binding sites were blocked with dried milk powder in PBS/0.1% Tween. The antibodies used were rabbit polyclonal anti-FcεRI α-chain (Upstate), rabbit polyclonal anti-FcεRI γ-subunit (Abcam), or mouse monoclonal anti-actin. As secondary antibodies HRP-conjugated anti-rabbit antibody (Amersham Biosciences) or HRP-conjugated anti-mouse antibody (Jackson Immunoresearch Laboratories) were used. Bands were detected with the SuperSignal CL-HRP Substrate system (Pierce, Rockford, IL) and quantified by densitometry (EasyWin, Herolab, Wiesloch, Germany).

## References

[1] Brandtzaeg P (1996). History of oral tolerance and mucosal immunity.. Ann N Y Acad Sci.

[2] Gounni AS, Lamkhioued B, Ochiai K, Tanaka Y, Delaporte E (1994). High-affinity IgE receptor on eosinophils is involved in defence against parasites.. Nature.

[3] Gurish MF, Bryce PJ, Tao H, Kisselgof AB, Thornton EM (2004). IgE enhances parasite clearance and regulates mast cell responses in mice infected with Trichinella spiralis.. J Immunol.

[4] Huber A, Genser D, Spitzauer S, Scheiner O, Jensen-Jarolim E (1998). IgE/anti-IgE immune complexes in sera from patients with Crohn's disease do not contain food-specific IgE.. Int Arch Allergy Immunol.

[5] van Spreeuwel JP, Lindeman J, van Maanen J, Meyer CJ (1984). Increased numbers of IgE containing cells in gastric and duodenal biopsies. An expression of food allergy secondary to chronic inflammation?. J Clin Pathol.

[6] De Lazzari F, Mancin O, Plebani M, Venturi C, Battaglia G (1994). High IgE serum levels and “peptic” ulcers: clinical and functional approach.. Ital J Gastroenterol.

[7] Gould HJ, Mackay GA, Karagiannis SN, O'Toole CM, Marsh PJ (1999). Comparison of IgE and IgG antibody-dependent cytotoxicity in vitro and in a SCID mouse xenograft model of ovarian carcinoma.. Eur J Immunol.

[8] Riemer AB, Untersmayr E, Knittelfelder R, Duschl A, Pehamberger H (2007). Active induction of tumor-specific IgE antibodies by oral mimotope vaccination.. Cancer Res.

[9] Jensen-Jarolim E, Achatz G, Turner MC, Karagiannis S, Legrand F (2008). AllergoOncology: the role of IgE-mediated allergy in cancer.. Allergy.

[10] Metzger H (1992). The receptor with high affinity for IgE.. Immunol Rev.

[11] Wan T, Beavil RL, Fabiane SM, Beavil AJ, Sohi MK (2002). The crystal structure of IgE Fc reveals an asymmetrically bent conformation.. Nat Immunol.

[12] Kinet JP (1999). The high-affinity IgE receptor (Fc epsilon RI): from physiology to pathology.. Annu Rev Immunol.

[13] Hakimi J, Seals C, Kondas JA, Pettine L, Danho W (1990). The alpha subunit of the human IgE receptor (FcERI) is sufficient for high affinity IgE binding.. J Biol Chem.

[14] Kuster H, Zhang L, Brini AT, MacGlashan DW, Kinet JP (1992). The gene and cDNA for the human high affinity immunoglobulin E receptor beta chain and expression of the complete human receptor.. J Biol Chem.

[15] Lin S, Cicala C, Scharenberg AM, Kinet JP (1996). The Fc(epsilon)RIbeta subunit functions as an amplifier of Fc(epsilon)RIgamma-mediated cell activation signals.. Cell.

[16] Kraft S, Kinet JP (2007). New developments in FcepsilonRI regulation, function and inhibition.. Nat Rev Immunol.

[17] Conrad DH (1990). Fc epsilon RII/CD23: the low affinity receptor for IgE.. Annu Rev Immunol.

[18] Gonzalez-Molina A, Spiegelberg HL (1976). Binding of IgE myeloma proteins to human cultured lymphoblastoid cells.. J Immunol.

[19] Boltz-Nitulescu G, Bazin H, Spiegelberg HL (1981). Specificity of fc receptors for IgG2a, IgG1/IgG2b, and IgE on rat macrophages.. J Exp Med.

[20] Grangette C, Gruart V, Ouaissi MA, Rizvi F, Delespesse G (1989). IgE receptor on human eosinophils (FcERII). Comparison with B cell CD23 and association with an adhesion molecule.. J Immunol.

[21] Kaiserlian D, Lachaux A, Grosjean I, Graber P, Bonnefoy JY (1993). Intestinal epithelial cells express the CD23/Fc epsilon RII molecule: enhanced expression in enteropathies.. Immunology.

[22] Yang PC, Berin MC, Yu LC, Conrad DH, Perdue MH (2000). Enhanced intestinal transepithelial antigen transport in allergic rats is mediated by IgE and CD23 (FcepsilonRII).. J Clin Invest.

[23] Yu LC, Yang PC, Berin MC, Di Leo V, Conrad DH (2001). Enhanced transepithelial antigen transport in intestine of allergic mice is mediated by IgE/CD23 and regulated by interleukin-4.. Gastroenterology.

[24] Bevilacqua C, Montagnac G, Benmerah A, Candalh C, Brousse N (2004). Food allergens are protected from degradation during CD23-mediated transepithelial transport.. Int Arch Allergy Immunol.

[25] Liu FT, Albrandt K, Mendel E, Kulczycki A, Orida NK (1985). Identification of an IgE-binding protein by molecular cloning.. Proc Natl Acad Sci U S A.

[26] Dumic J, Dabelic S, Flogel M (2006). Galectin-3: an open-ended story.. Biochim Biophys Acta.

[27] Moutsatsos IK, Wade M, Schindler M, Wang JL (1987). Endogenous lectins from cultured cells: nuclear localization of carbohydrate-binding protein 35 in proliferating 3T3 fibroblasts.. Proc Natl Acad Sci U S A.

[28] Woo HJ, Shaw LM, Messier JM, Mercurio AM (1990). The major non-integrin laminin binding protein of macrophages is identical to carbohydrate binding protein 35 (Mac-2).. J Biol Chem.

[29] Castronovo V, Campo E, van den Brule FA, Claysmith AP, Cioce V (1992). Inverse modulation of steady-state messenger RNA levels of two non-integrin laminin-binding proteins in human colon carcinoma.. J Natl Cancer Inst.

[30] Jensen-Jarolim E, Gscheidlinger R, Oberhuber G, Neuchrist C, Lucas T (2002). The constitutive expression of galectin-3 is downregulated in the intestinal epithelia of Crohn's disease patients, and tumour necrosis factor alpha decreases the level of galectin-3-specific mRNA in HCT-8 cells.. Eur J Gastroenterol Hepatol.

[31] Byrd JC, Bresalier RS (2004). Mucins and mucin binding proteins in colorectal cancer.. Cancer Metastasis Rev.

[32] Borkowski TA, Jouvin MH, Lin SY, Kinet JP (2001). Minimal requirements for IgE-mediated regulation of surface Fc epsilon RI.. J Immunol.

[33] Kalesnikoff J, Huber M, Lam V, Damen JE, Zhang J (2001). Monomeric IgE stimulates signaling pathways in mast cells that lead to cytokine production and cell survival.. Immunity.

[34] Stokes AJ, Shimoda LM, Lee JW, Rillero C, Chang YT (2006). Fcepsilon RI control of Ras via inositol (1,4,5) trisphosphate 3-kinase and inositol tetrakisphosphate.. Cell Signal.

[35] Repasky GA, Chenette EJ, Der CJ (2004). Renewing the conspiracy theory debate: does Raf function alone to mediate Ras oncogenesis?. Trends Cell Biol.

[36] Hershberg RM, Framson PE, Cho DH, Lee LY, Kovats S (1997). Intestinal epithelial cells use two distinct pathways for HLA class II antigen processing.. J Clin Invest.

[37] Blumberg RS, Lencer WI, Zhu X, Kim HS, Claypool S (1999). Antigen presentation by intestinal epithelial cells.. Immunol Lett.

[38] Maurer D, Fiebiger S, Ebner C, Reininger B, Fischer GF (1996). Peripheral blood dendritic cells express Fc epsilon RI as a complex composed of Fc epsilon RI alpha- and Fc epsilon RI gamma-chains and can use this receptor for IgE-mediated allergen presentation.. J Immunol.

[39] Maurer D, Fiebiger E, Reininger B, Ebner C, Petzelbauer P (1998). Fc epsilon receptor I on dendritic cells delivers IgE-bound multivalent antigens into a cathepsin S-dependent pathway of MHC class II presentation.. J Immunol.

[40] Negrao-Correa D, Adams LS, Bell RG (1996). Intestinal transport and catabolism of IgE: a major blood-independent pathway of IgE dissemination during a Trichinella spiralis infection of rats.. J Immunol.

[41] Ramaswamy K, Goodman RE, Bell RG (1994). Cytokine profile of protective anti-Trichinella spiralis CD4+ OX22− and non-protective CD4+ OX22+ thoracic duct cells in rats: secretion of IL-4 alone does not determine protective capacity.. Parasite Immunol.

[42] Gscheidlinger R (1998). IgE-binding structures in human intestinal epithelia..

[43] Seminario MC, Saini SS, MacGlashan DW, Bochner BS (1999). Intracellular expression and release of Fc epsilon RI alpha by human eosinophils.. J Immunol.

[44] Delespesse G, Sarfati M, Wu CY, Fournier S, Letellier M (1992). The low-affinity receptor for IgE.. Immunol Rev.

[45] Ayabe T, Satchell DP, Wilson CL, Parks WC, Selsted ME (2000). Secretion of microbicidal alpha-defensins by intestinal Paneth cells in response to bacteria.. Nat Immunol.

[46] Wehkamp J, Stange EF (2006). Paneth cells and the innate immune response.. Curr Opin Gastroenterol.

[47] Shen B, Porter EM, Reynoso E, Shen C, Ghosh D (2005). Human defensin 5 expression in intestinal metaplasia of the upper gastrointestinal tract.. J Clin Pathol.

[48] Wada R, Yamaguchi T, Tadokoro K (2005). Colonic Paneth cell metaplasia is pre-neoplastic condition of colonic cancer or not?. J Carcinog.

[49] Schmidt GH, Wilkinson MM, Ponder BA (1985). Cell migration pathway in the intestinal epithelium: an in situ marker system using mouse aggregation chimeras.. Cell.

[50] Gregorieff A, Pinto D, Begthel H, Destree O, Kielman M (2005). Expression pattern of Wnt signaling components in the adult intestine.. Gastroenterology.

[51] Bises G, Kallay E, Weiland T, Wrba F, Wenzl E (2004). 25-hydroxyvitamin D3-1alpha-hydroxylase expression in normal and malignant human colon.. J Histochem Cytochem.

[52] Cross HS, Bises G, Lechner D, Manhardt T, Kallay E (2005). The Vitamin D endocrine system of the gut–its possible role in colorectal cancer prevention.. J Steroid Biochem Mol Biol.

